# Temperature Compensation for MEMS Accelerometer Based on a Fusion Algorithm

**DOI:** 10.3390/mi15070835

**Published:** 2024-06-27

**Authors:** Yangyanhao Guo, Zihan Zhang, Longkang Chang, Jingfeng Yu, Yanchao Ren, Kai Chen, Huiliang Cao, Huikai Xie

**Affiliations:** 1 Key Laboratory of Instrumentation Science & Dynamic Measurement, Ministry of Education, North University of China, Taiyuan 030051, China; s202206044@st.nuc.edu.cn; 2 School of Information and Software Engineering, University of Electronic Science and Technology of China, Chengdu 610054, China; 2021090919020@std.uestc.edu.cn; 3 College of Intelligence Science and Technology, National University of Defense Technology, Changsha 410073, China; 4 Quanzhou Yunjian Measurement Control and Perception Technology Innovation Research Institute, Quanzhou 362000, China; yujingfeng@yjqz.org (J.Y.); renyanchao@yjqz.org (Y.R.); 5 School of Automation Engineering, University of Electronic Science and Technology of China, Chengdu 610054, China; kaichen@uestc.edu.cn; 6 Chongqing Institute of Microelectronics & Microsystems, Beijing Institute of Technology, Chongqing 400000, China; hk.xie@bit.edu.cn

**Keywords:** accelerometer, denoising, temperature compensation

## Abstract

This study proposes a fusion algorithm based on forward linear prediction (FLP) and particle swarm optimization–back propagation (PSO-BP) to compensate for the temperature drift. Firstly, the accelerometer signal is broken down into several intrinsic mode functions (IMFs) using variational modal decomposition (VMD); then, according to the FE algorithm, the IMF signal is separated into mixed components, temperature drift, and pure noise. After that, the mixed noise is denoised by FLP, and PSO-BP is employed to create a model for temperature adjustment. Finally, the processed mixed noise and the processed IMFs are rebuilt to obtain the enhanced output signal. To confirm that the suggested strategy works, temperature experiments are conducted. After the output signal is processed by the VMD-FE-FLP-PSO-BP algorithm, the acceleration random walk has been improved by 23%, the zero deviation has been enhanced by 24%, and the temperature coefficient has been enhanced by 92%, compared with the original signal.

## 1. Introduction

Micro-electro-mechanical systems (MEMS) accelerometers present the advantages of MEMS technology, including low cost, small power consumption, and wide application, including in healthcare, Earth exploration, and consumer electronics [1,2]. 

Due to the influence of the accelerometer itself and the signal hardware acquisition circuit, the collected accelerometer signal contains a large amount of noise. Directly analyzing the output signal inevitably leads to errors and requires corresponding denoising processing. Traditional denoising methods include wavelet transform, wavelet thresholding, and forward linear prediction, among others. Chang proposed a parallel method in which forward linear prediction is used to denoise mixed features [3]. However, traditional algorithms not only eliminate noise but also suppress useful signals. Based on this, empirical modal decomposition (EMD) [4], EEMD, and other algorithms have been proposed. Due to the problem of modal aliasing in EMD, Wang et al. utilize CEEMDAN to decompose the signal of the fiber optic gyroscope and perform noise suppression and temperature compensation [5]. The output of MEMS gyroscopes is decomposed by interval local mean decomposition and time–frequency peak filtering is used to denoise the mixed component [6]. A new decomposition algorithm, which is variational modal decomposition (VMD), was first proposed in 2014 and in [7]; therefore, combining VMD with forward linear prediction is an effective method for suppressing noise.

Due to the influence of the materials and fabrication, MEMS accelerometers’ performance degrades dramatically in a temperature-changing environment, which greatly limits their applications [8]. In recent years, extensive literature has been proposed to address the thermal behavior of MEMS accelerometers [9]. Generally, the main ways to suppress temperature drift include hardware structures and software processing.

Hardware compensation generally aims at optimizing the MEMS accelerometer structure, circuit control, and the accelerometer’s working environment. Liu suppresses temperature compensation by modeling parasitic resistance [10]. Wang has improved both the structural design (adding electrostatic softening springs) and the control circuit (using continuous damping technology) to enhance the measurement accuracy of the accelerometer [11]. Li suggested a unique differential silicon substrate that can successfully lower a device’s temperature drift [12]. He et al. first established analytical models for bias temperature drift (TDB) and scaling factor temperature drift (TDMF) and then designed a temperature model to compensate for TDB and TDSF [13]. However, hardware compensation not only has a complicated process but also needs additional hardware.

A temperature drift compensation model is built into the software compensation to investigate the relationship between the temperature and the MEMS accelerometer’s output to prevent temperature drift [14]. Khankalantary modeled the dependence of error coefficients on the temperature and proposed an online calibration method [15]. The author achieves the thermal compensation of differential vibration accelerometers by identifying the frequency of the approximate linear drift caused by temperature changes [16]. The author uses a genetic algorithm to improve the estimation accuracy of the temperature drift error, thereby improving the speed of its TDE calculation [17]. The literature [18] proposes a strong tracking Kalman filter to address the issue of unknown IMU error models, which adapts to the model uncertainty of gyroscope and accelerometer errors through multiple fading factors. Establishing a temperature compensation model has many advantages that hardware compensation cannot match, such as low cost and great flexibility, so it has become a current trend. A cost-effective and useful temperature error correction model for MEMS accelerometers has been developed through the numerical analysis of test data. It is a component of a recent trend in research.

The modeling of temperature drift in MEMS accelerometers has been accomplished with success using the neural network, which is based on the evolutionary algorithm. Consequently, to build a more accurate temperature drift model for MEMS accelerometers, a modeling method based on PSO-BP is proposed, and we propose a fusion algorithm, where denoising and temperature compensation are processed in parallel.

The specific organization of this paper is arranged as follows: Section 2 introduces the hardware structure of the accelerometer. A fusion algorithm is described in Section 3. Section 4 shows the experimental results and an analysis result of different algorithms. Section 5 gives the conclusions.

## 2. Structure of MEMS Accelerometer

In this paper, the signals collected come from the laboratory self-research monolithic capacitive accelerometers. Since capacitive accelerometers have a guaranteed good device stability, sufficient range, small influence of coupling between cross-axes, and lower standards for process requirements, they have the best overall performance and the widest range of practical applications and can be applied in high-precision fields.

**Figure 1:**
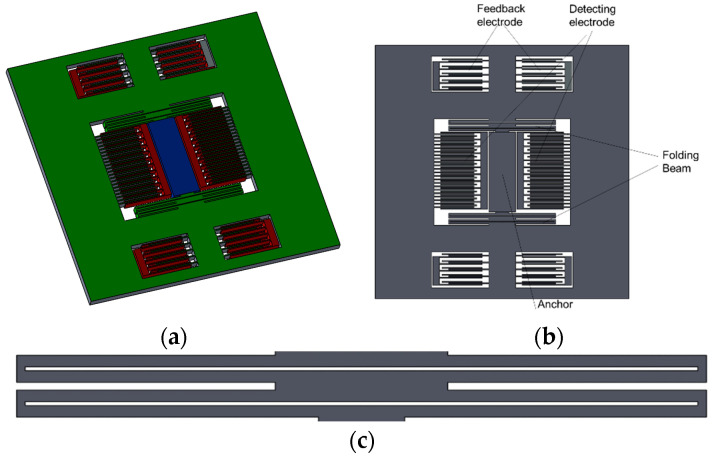
Structure of monolithic capacitive accelerometer. (**a**) Overall 3D model, (**b**) planar structure, (**c**) return beam structure.

To improve the structural utilization and decrease the effects of thermo-mechanical stress on the sensitive structure, the accelerometer is structurally designed in a very compact form, and the anchor point is fixed in the center of the structure. The operating mode is fully differential comb capacitive detection with uniform fixed teeth and electrostatic feedback, with a closed-loop operation up to a maximum closed loop, which reaches 15 g. The use of a girder structure makes the accelerometer more compact, which can make the stiffness moderate and enhance the release of residual stress. In the closed-loop operation, the layout of the calibration and feedback combs also ensures that the structure is stable. The structure of this capacitive accelerometer is shown in Figure 1.

**Figure 2:**
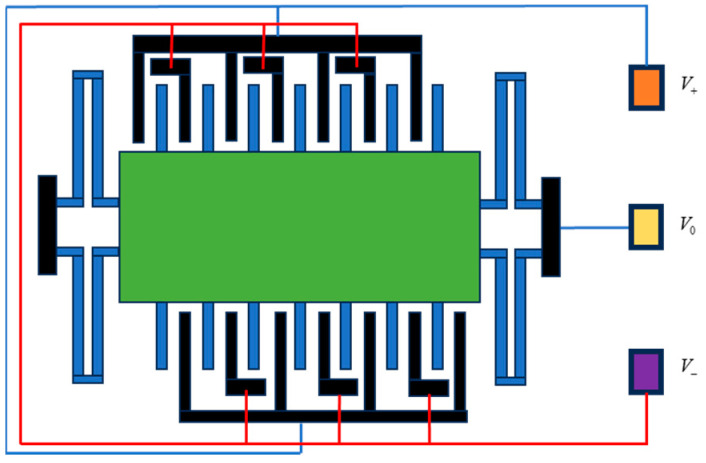
Differential comb capacitance detection model.

The principle of this accelerometer is to detect acceleration using a differential capacitance detection circuit and electrostatic force-feedback closed loop. In the ideal case, when the input of the accelerometer is zero, the active mass piece is located in the middle of the electrode plate (i.e., in the zero position). When the acceleration input is not zero, the active mass piece of the electrostatic force on the side with an increased gap will be in motion, i.e., to produce the corresponding direction of displacement because of the role of the closed-loop system of negative feedback. In the closed-loop system, when the system parameters are properly adjusted, an overall positive feedback effect in the open loop will not be formed, and the active pole plate is always near zero. The model of this accelerometer is shown in Figure 2.

**Figure 3:**
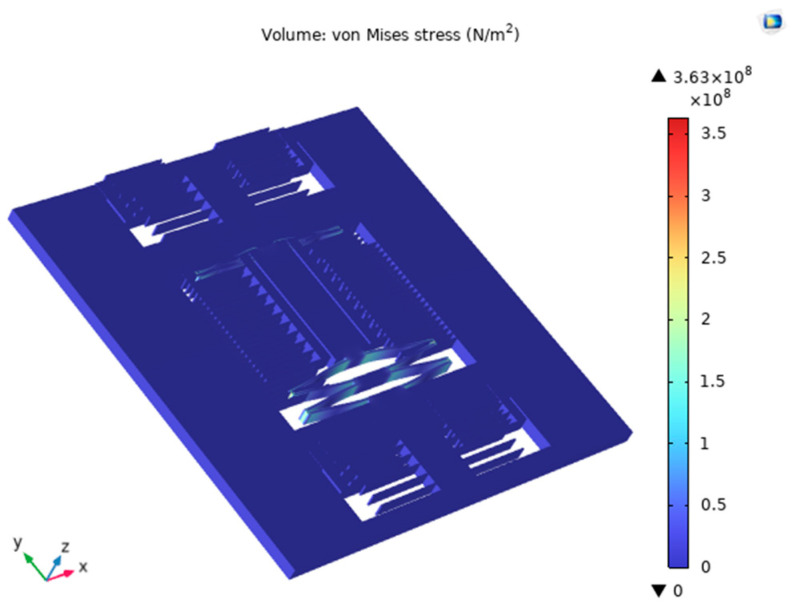
Stress diagram of the horizontal axial structure under 2000 g 11 ms shock.

During the testing process, since repeated impact experiments can cause fatigue injury to the chip structure, reducing the fracture strength of the silicon material, ultimately leading to sensor damage in severe cases, finite element analysis (FEA), as shown in Figure 3 (which represents its structural stresses in the horizontal and vertical axial directions under a 2000 g 11 ms impact), is required to optimize the sensor’s structural parameters (Table 1).

**Figure 4:**
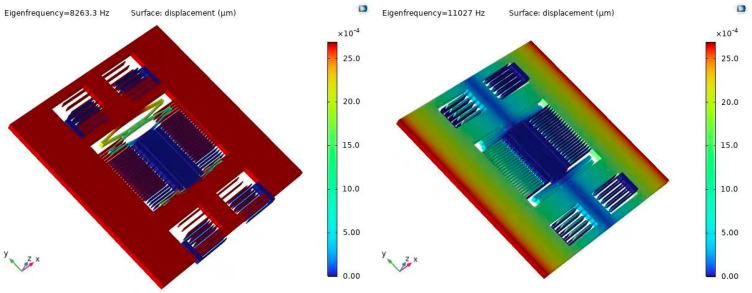

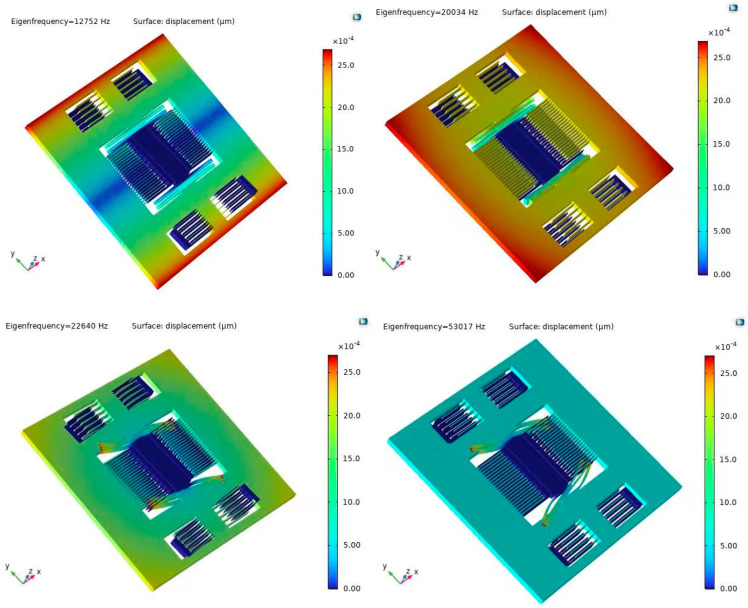
First six orders of modal shapes of the accelerometer.

To acquire the vibration characteristics of the sensor, we perform a modal analysis including the intrinsic frequency (Table 2), modal vibration pattern (Figure 4), and vibration stability. The first-order modes in Figure 4 are the operating modes for detecting the horizontal axis acceleration with a first-order intrinsic frequency of 8595.3 Hz, which provides a wide baseband for the accelerometer. The rest of the modes are interference modes, and the significant disparity between working modes and interference modes can effectively avoid coupled vibration and improve the stability of the sensor structure.

**Figure 5:**
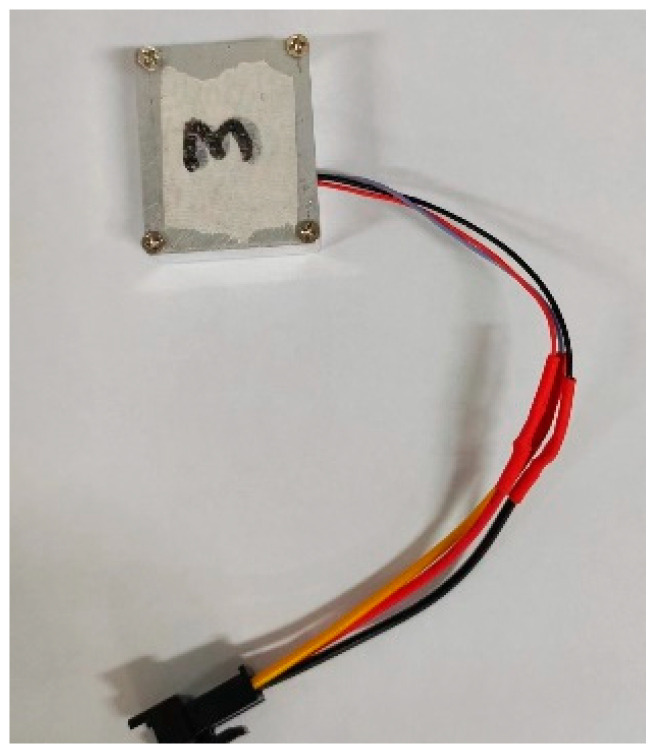
The overall package of the accelerometer.

The whole image of the accelerometer is shown in Figure 5. The monolithic capacitive accelerometer prototype prepared on silicon has a horizontal axial closed-loop maximum of 15 g, an axial sensitivity of 65 mV/g, an intrinsic frequency of 8263.3 Hz, respectively, an accelerometer quality factor of 8.54 at non-vacuum atmospheric pressure, and a resistance to overloading of 2000 g, which has been verified by a Hopkinson bar.

## 3. Parallel Processing Methods

### 3.1. Variational Mode Decomposition (VMD)

VMD non-recursively decomposes a real signal with multiple frequency components into multiple eigenmode functions *u_k_* and obtains the one-sided spectrum of each *u_k_*, while tuning the frequency of each *u_k_*, and finally adds constraints to obtain a constrained variational model [19]:

(1) $$\begin{array}{c} \left\{\underset{\left\{u_{k}\right\}\left\{\omega_{k}\right\}}{\min}\left\{{\sum_{K}^{k=1}\;\partial_{t}\left[\left(\delta \left(t\right)+\frac{j}{\pi t}\right)\times u_{k}\left(t\right)\right]e^{-j\omega_{k}t}}_{2}^{2}\right\}\right.\\ s.t\;\sum_{K}^{k}\;u_{k}=f \end{array}$$

where $\omega_{k}$ is the frequency center of each order modal component obtained by assuming decomposition; *u_k_* is the *k*-th IMF modal component; *f* is the original signal; $\delta \left(t\right)$ is an impulse function.

The above problem is transformed into a no-approximation by means of a Lagrangian function bounded variational problem [20]:

(2) $$\begin{array}{cc} L_{\left(u_{k},\omega_{k},\lambda \right)}= & {\alpha \sum_{K}^{k=1}\;\partial_{t}\left[\left(\delta \left(t\right)+\frac{j}{\pi t}\right)\times u_{k}\left(t\right)\right]e^{-j\omega_{k}t}}_{2}^{2}\\ & +\;f\left(t\right)-{\sum_{K}^{k=1}\;u_{k}\left(t\right)}_{2}^{2}+\langle \lambda \left(t\right),\;f\left(t\right)-\sum_{K}^{k=1}\;u_{k}\left(t\right)\rangle \end{array}$$

where α is the penalty factor; λ is the Lagrange multiplier. Then, the alternating multiplier direction algorithm is employed to solve and update the center of each IMF center frequency and bandwidth of each IMF component:

(3) $$\omega_{k}^{n+1}=\frac{\int_{0}^{\infty }\omega {\left|{\hat{u}}_{k}\left(\omega \right)\right|}^{2}d\omega }{\int_{0}^{\infty }{\left|{\hat{u}}_{k}\left(\omega \right)\right|}^{2}d\omega }$$

(4) $${\hat{u}}_{k}^{n+1}\left(\omega \right)=\frac{\hat{f}\left(\omega \right)-\sum_{i\neq k}{\hat{u}}_{i}\left(\omega \right)+(\hat{\lambda }(\omega )/2)}{1+2\alpha {\left(\omega -\omega_{k}\right)}^{2}}$$

where ${\hat{u}}_{k}^{n+1}\left(\omega \right)$ is the filtering result of the current residual $\hat{f}\left(\omega \right)-\sum_{i\neq k}{\hat{u}}_{k}^{n+1}\left(\omega \right)$; $\omega_{k}^{n+1}$ is the power spectrum center of the *k*-th IMF.

From the above equations, it can be seen that the parameter *K* and α taking values will have a great influence on the arithmetic decomposition results of the algorithm. Too small a value of *K* will lead to insufficient decomposition, while too large a value of *K* will easily lead to problems such as false components and frequency overlapping. If the value of α is too small, the signal denoising is not thorough enough, and if it is too large, the effective components will be removed incorrectly. Empirical selection of the above parameter values cannot ensure that they are optimal.

To solve this problem, the author introduced the Aquila optimization algorithm to improve the VMD and obtain the best parameter combination [*K*, *α*]. In this process, the envelope entropy reflects the sparsity of the signal; the more noise in the signal, the less effective components, and the larger the envelope entropy. On the contrary, the more active components a signal contains, the smaller the envelope entropy. In other words, when the envelope entropy is the lowest, the signal contains the most effective components, and the corresponding parameters are optimal. Therefore, the minimum value of the envelope entropy is employed as the fitness function of the Skyhawk optimizer to evaluate the decomposition effect of the parameter combination. The mathematical calculation formula of the envelope entropy *E_p_* is

(5) $$E_{p}=-\sum_{m}^{q=1}\;p_{q}\mathrm{lg}p_{q}$$

(6) $$p_{q}=a\left(q\right)/\sum_{m}^{q=1}\;a\left(q\right)$$

(7) $$a\left(q\right)=\sqrt{{\left[x(q)\right]}^{2}+{\left\{H[x\left(q\right)]\right\}}^{2}}$$

where *m* is the number of sampling points; *p_q_* is the normalized form of *a*(*q*); *a*(*q*) is the envelope signal transformed by Hilbert [21].

### 3.2. Fuzzy Entropy (FE)

The fuzzy entropy method based on fuzzy theory uses the membership function to calculate the fuzzy similarity between different hidden modes. Specifically, for a given time series $x_{\left(t\right)}$, t=1,2,…,T where *T* is the length of *x*_(*t*)_, its fuzzy entropy is calculated as follows [22]:

Step 1: the embedding vector *X*_(*i*)_ is structured, the embedding dimension is *m*, and the formula is

(8) $$x_{i}^{m}=\left[x_{\left(i\right)},x_{\left(i+1\right)},\cdots ,x_{\left(i+m-1\right)}\right],\;\;1\leq i\leq T-m+1$$

Step 2: $d_{ij}^{m}$ is the distance between $X_{\left(i\right)}$ and $X_{\left(j\right)}$:

(9) $$d_{ij}^{m}=d\left[X_{i}^{m},X_{j}^{m}\right]=\underset{k=0,1,\cdots ,m-i}{\max}\;\left\{|\left[x_{\left(i+k\right)}-x_{0\left(i\right)}\right]-\left[x_{\left(j+k\right)}-x_{0\left(j\right)}\right]|\right\}$$

Step 3: the similarity between $X_{\left(i\right)}$ and $X_{\left(j\right)}$, which is $D_{ij}^{m}$, is calculated as follows:

(10) $$D_{ij}^{m}(n,r)=u\left(d_{ij}^{m},n,r\right)$$

where $u\left(d_{ij}^{m},n,r\right)$ is a fuzzy membership function.

Step 4: $\varphi_{\left(n,r\right)}^{m}$ is defined by $D_{ij}^{m}$:

(11) $$\varphi^{m}=\frac{1}{T-m+1}\underset{T-m+1}{\sum^{i=1}}\left(\frac{1}{T-m}\underset{T-m+1}{\sum^{j=1,\;i\neq j}}D_{ij}^{m}\right)$$

Step 5: similarly, construct the vector, and $\varphi^{m+1}$ is computed.

(12) $$\varphi_{\left(n,r\right)}^{m+1}=\frac{1}{T-m}\underset{T-m+1}{\sum^{i=1}}\left(\frac{1}{T-m-1}\underset{T-m}{\sum^{j=1,\;i\neq j}}\;D_{ij}^{m+1}\right)$$

Step 6: the value of the entropy of time series *x*_(*t*)_ is calculated based on the Equation (13):

(13) $${\mathrm{FE}}_{\left(m,n,r,T\right)}=\ln\varphi_{(n,r)}^{m}-\ln\varphi_{(n,r)}^{m+1}$$

### 3.3. Forward Linear Prediction (FLP)

This algorithm predicts the signal at time *t* by multiplying the signal before time t with the set weight. In applications, the initial weight value is usually set to 0, and then through iteration, the minimum mean square error theory is used to minimize the difference between the current time and the predicted value, ultimately obtaining a stable and convergent weight value [23].

The estimated value of the output data at time *t* is

(14) $$\hat{x}\left(t\right)=\sum_{p=1}^{K}a_{p}x\left(t-p\right)=A^{T}X\left(t-1\right)$$

In the equation, *x*(*t* − *p*) is the accelerometer output data before time *t*; matrix *A* is the weight coefficient vector of the prediction filter; *a_p_* is the weight; *K* is the order. In FLP filtering, the selection of *K* has a significant impact on the filtering effect, and the vector before time *t* composed of *K* is shown below [24]:

(15) $$X\left(t-1\right)={\left\{x\left(t-1\right),x\left(t-2\right),\ldots ,x\left(t-K\right)\right\}}^{T}$$

According to the minimum mean square error (MSE) criterion, the MSE of FLP filtering is defined as

(16) $$J\left(t\right)=E\left[e^{2}\left(t\right)\right]$$

In the equation, $e\left(t\right)=x\left(t\right)-\hat{x}\left(t\right)$. By using *e*(*t*) to select appropriate weight values, the adjustment of weights is

(17) $$A\left(t\right)=A\left(t-1\right)+ve\left(t\right)X\left(t-1\right)$$

In the equation, *v* is a positive constant, adjusted by *v*, and the value of *v* can adaptively adjust the convergence speed of the FLP filtering process.

### 3.4. The PSO-BP Method

PSO is a swarm intelligence optimization method that originates from the predatory behavior of animals in the biological world. Similar to genetic algorithms, particle swarm optimization also uses the fitness of individuals in the population to evaluate their strengths and weaknesses, but without the crossover and mutation operations of genetic operations. A group of particles are initialized in the weight and threshold solvable space of the BP method, each including threshold data and the ownership value of the BP neural network. The position, velocity, and fitness values of the particles within the population define the particle characteristics. The ideal BP neural network initialization weight and threshold are found by tracking the best individual and population positions, which can quicken the BP neural network’s rate of convergence and enhance its predictive capabilities [25].

In the PSO, each iteration of particles updates their velocity and position through individual and global extremum values. The updated equation is as follows:

The speed update formula is

(18) $$v_{i}^{k+1}=wv_{i}^{k}+c_{1}r_{1}\left(p_{g}^{k}-x_{i}^{k}\right)+c_{2}r_{2}\left(p_{z}^{k}-x_{i}^{k}\right)$$

The position update formula is

(19) $$x_{i}^{k+1}=x_{i}^{k}+v_{i}^{k+1}$$

where *k* is the current number of iterations; $V_{i}^{k+1}$ refers to the movement speed of the *i*-th particle in the population in the *k +* 1 th generation; $V_{i}^{k}$ refers to the *k*-th generation movement speed of the *i*-th particle; *w* is the inertia weight; *r*_1_ and *r*_2_ ∈ [0, 1]; *c*_1_ and *c*_2_ are the acceleration value of particles, taken as non-negative constants.

Select the variable inertia weights, with the reference formula as

(20) $$w=w_{\max}-\frac{w_{\max}-w_{\min}}{i_{\max}}i_{t}$$

In the formula, *i_max_* refers to the maximum number of iterations; *w_max_* represents the weight of the maximum inertia; *w_min_* represents the minimum inertia weight. When the maximum velocity of a particle is very small, *w* is generally made close to 1. Conversely, *w* is often taken as 0.8. When *w* is small, it is beneficial to utilize the local search ability of the PSO method; when *w* is large, it focuses on utilizing its global search ability.

**Figure 6:**
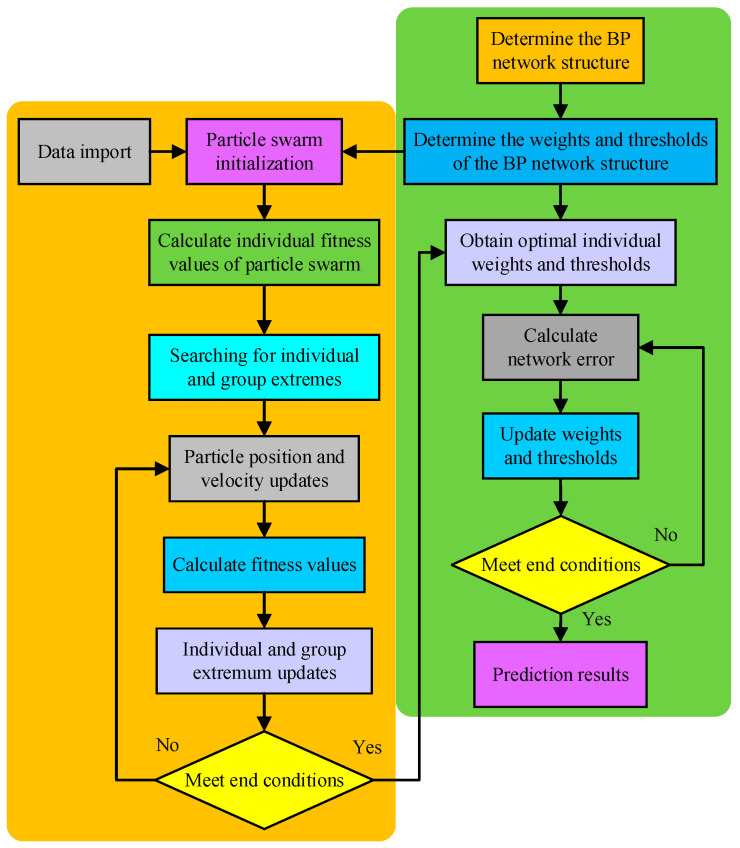
PSO-BP algorithm flowchart.

The flowchart of the PSO-BP algorithm is shown in Figure 6. Compared to the GA algorithm, the particle swarm initialization does not use encoding, but randomly initializes the particle positions and velocities within a certain range based on the connection weights and total threshold values between each layer. The fitness function also uses the fitness function in GA-BP to iteratively search for individual and population extremes and uses the population extremes (including BP neural network ownership values and thresholds) as the initial weights and thresholds for BP neural network training and prediction, improving the prediction performance.

### 3.5. A Fusion Algorithm

In this paper, a fusion algorithm based on VMD-FE-FLP-PSO-BP is expressed as follows:

Step 1: the original signal is acquired by the temperature experiment.

Step 2: employing VMD to decompose original data into IMFs and the FE algorithm is employed to classify IMFs.

After obtaining the initial MEMS accelerometer data, the temperature drift sequence is decomposed using VMD. The entropy of the sequence is then determined using FE. The decomposed IMFs are classified into three signal components based on their autocorrelation and complexity: noise signals, mixed signals, and temperature drift signals.

Step 3: using FLP to denoise the mixed component, the PSO-BP algorithm is used to establish the temperature model.

Since there is a lot of white noise in the noise signal and very few useful components, it is eliminated, and the temperature. The drift signal is the accelerometer output with temperature, which is then maintained and the component is investigated. The algorithm’s processing of mixed signals, which are a combination of noise and useful components, is crucial. FLP is used to filter the mixed signals and the drift components are modeled by the PSO-BP algorithm. Subsequently, the output of the accelerometer is acquired by reconstructing the drift and mixed signals following filtering.

Step 4: construct the signal after the above steps.

**Figure 7:**
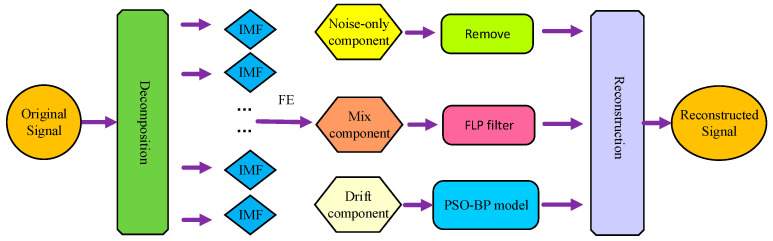
VMD-FE-FLP-PSO-BP algorithm flowchart.

The steps of VMD-FE-FLP-PSO-BP are shown in Figure 7.

## 4. Experiment and Results

### 4.1. Acc Temperature Experiment

**Figure 8:**
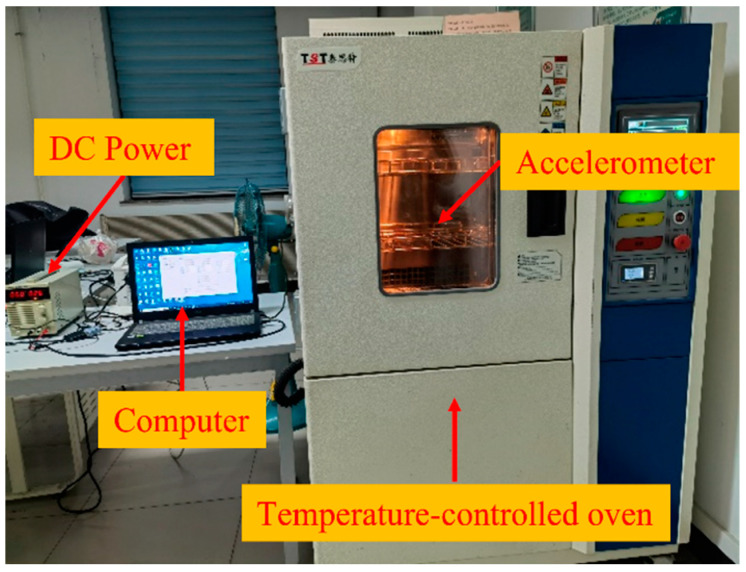
Temperature experiment equipment.

**Figure 9:**
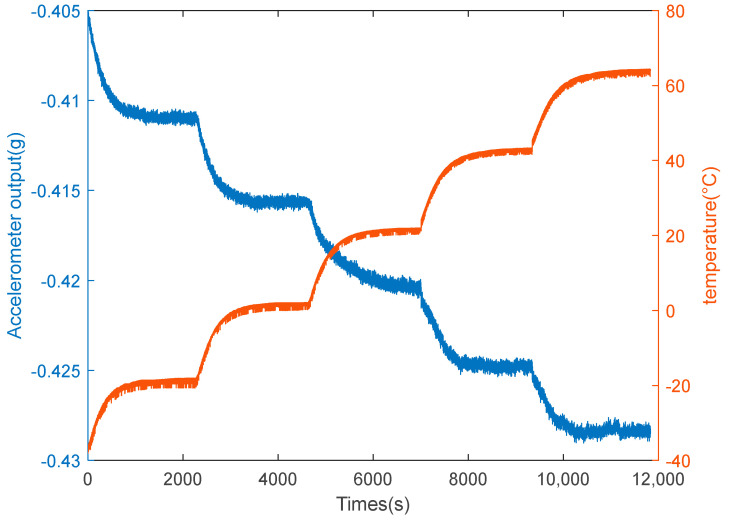
Experimental results.

As illustrated in Figure 8, we used a Gwinstek GPS-4303C DC power supply which is come from TEquipment in Long Blanche, New Jersey and sourced from China to conduct temperature experiments to assess the monolithic capacitive accelerometer’s temperature characteristics. To prevent motion from impacting the accelerometer, it was first mounted on a stationary plane. The output cable was then linked to a laptop and a Gwinstek DC power supply. Subsequently, the warm box’s temperature was adjusted between −30 °C and +60 °C. Ultimately, the accelerometer’s raw output signal was recorded, and the power supply was turned on. A temperature sensor, whose value was synchronized with the accelerometer output, was used to continuously capture data while monitoring the internal temperature of the metal container. Figure 9 displays the temperature experiment’s findings. It is evident that the temperature has a significant impact on the accelerometer’s output accuracy.

### 4.2. Result Analysis

**Figure 10:**
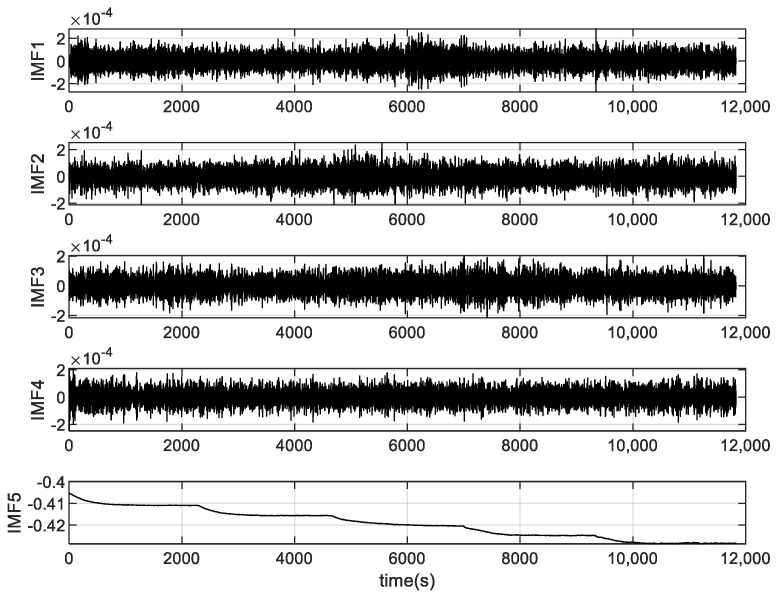
Intrinsic mode functions by VMD.

**Figure 11:**
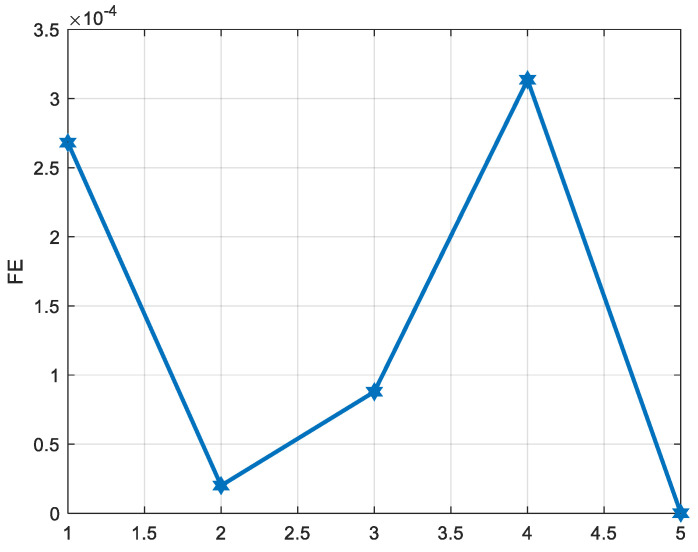
Feature components extraction.

**Figure 12:**
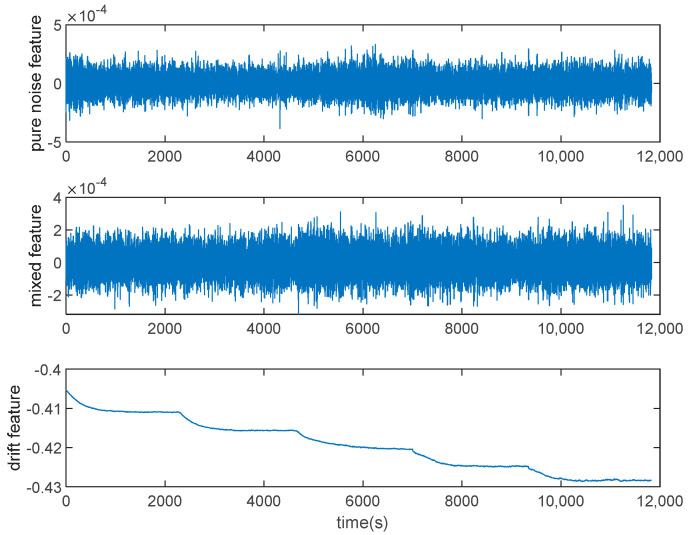
Components obtained from FE.

Firstly, we use the VMD algorithm to divide the output of the signal with five IMFs, and the exploded view is shown in Figure 10. If each IMF is processed, the computational cost is high and inaccurate, so it is necessary to use this entropy to classify modal functions. Therefore, the FE method is employed to compute the FE values of the IMFs. FE classifies the IMFs into three groups based on serial autocorrelation and complexity: drift component, mixture component, and noise component, which is displayed in Figure 11. From Figure 11, we consider IMF1 and IMF4 as noise components because the FE value of IMF1 and IMF4 is bigger than the other IMFs. IMF2 and IMF3 are considered as mixed components, while the drift component is IMF5. The results of the three components are shown in Figure 12. 

**Figure 13:**
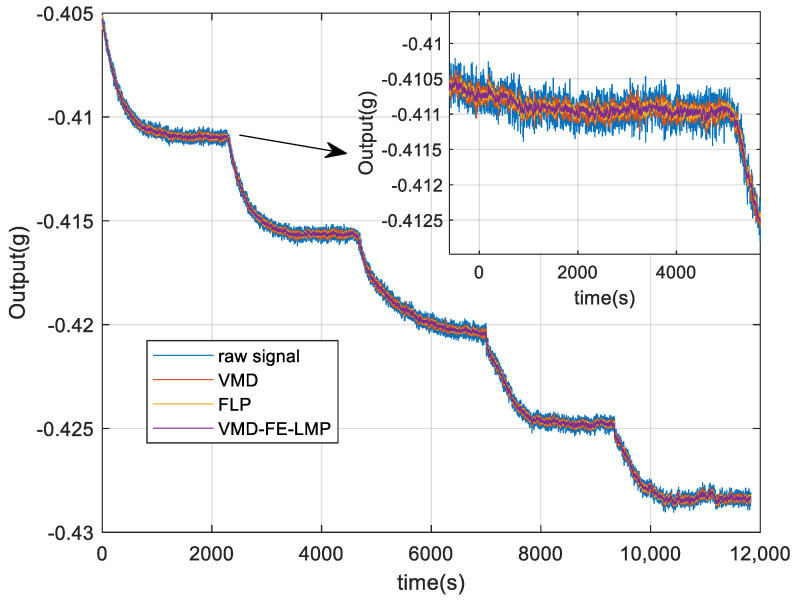
Signal reconstruction after denoising by different algorithms.

In this research, FLP is employed to filter the mixture component. The drift components are preserved, while the noise components are deleted. At last, the denoised reconstructed signal is created by reassembling the data, as illustrated in Figure 13. From Figure 13, the signal after denoising is obviously smoother than the original signal and it can be concluded that VMD and FLP have denoising ability and VMD-FE-FLP does a good job at suppressing noise. 

**Figure 14:**
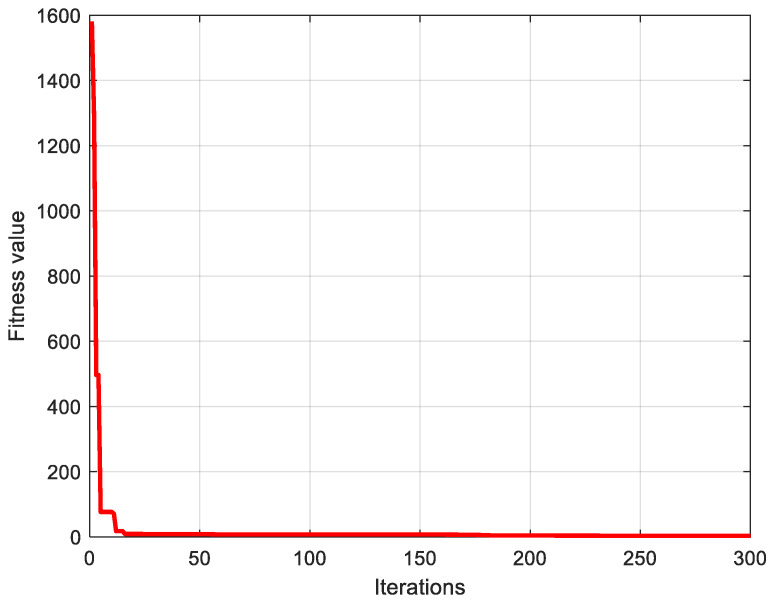
The cumulative error iteration curve.

**Figure 15:**
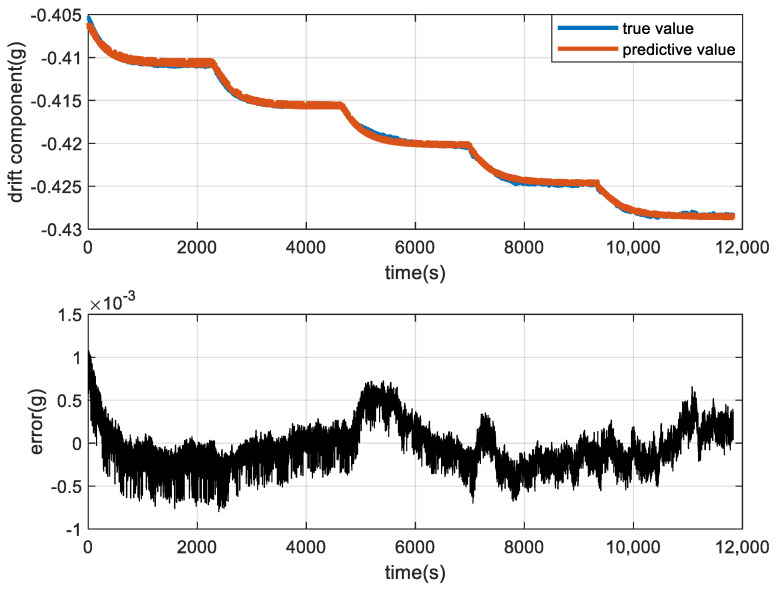
Predictive value by the PSO-BP algorithm and errors between the true value and predictive value.

Figure 14 shows the cumulative error iteration curve; as the number of iterations increases, the error becomes smaller and tends towards a stable value. Figure 15 shows the difference between the predicted and true values. From Figure 15, it can be seen that PSO-BP can precisely adjust for the accelerometer’s temperature drift signal and has a high forecast accuracy.

**Figure 16:**
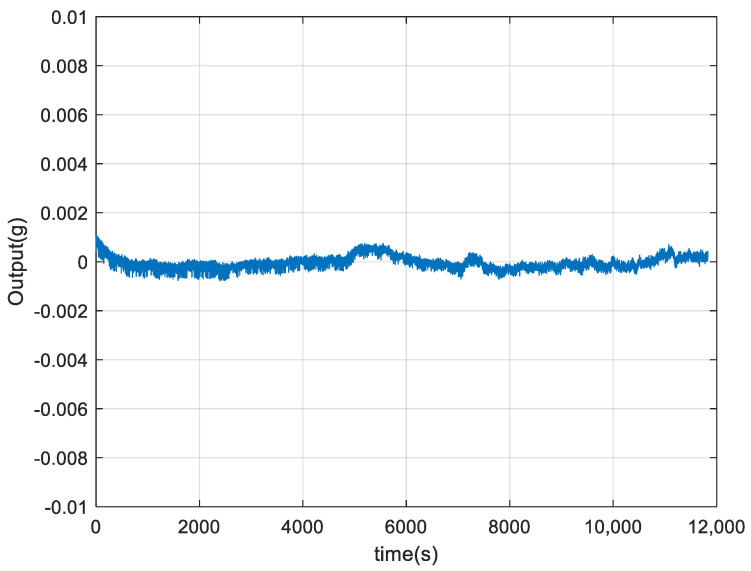
Output signal processed by the fusion algorithm.

Finally, the three components that are processed separately by the above steps are reconstructed to obtain the final signal, as shown in Figure 16. After the proposed fusion algorithm, we have calculated the temperature coefficient, which is reduced from 2.4 × 10^−4^ g/°C to 1.8 × 10^−5^ g/°C; the temperature character of the acceleration signal is improved obviously, which can prove the VMD-FE-FLP-PSO-BP algorithm is effective in reducing the influence of temperature drift.

**Figure 17:**
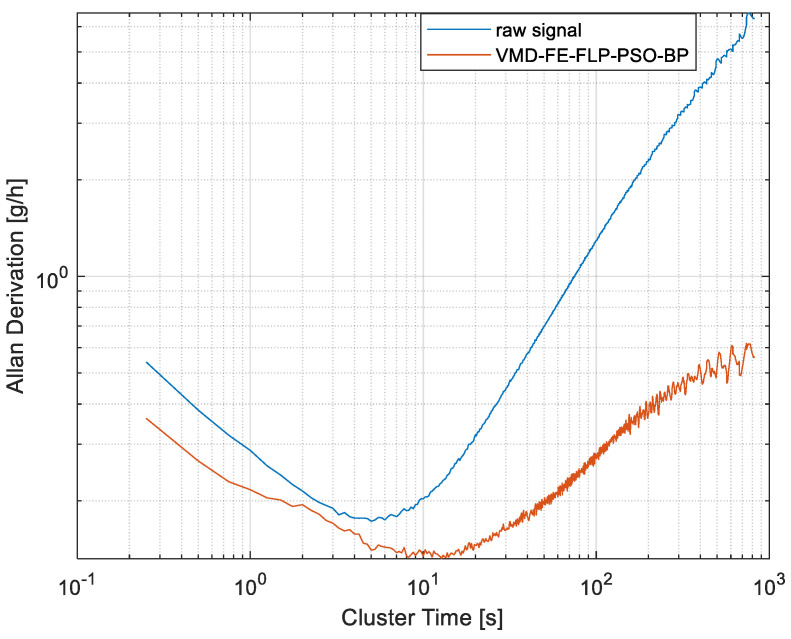
Allan variance curve comparison.

To qualify the performance indicators, Allan variance [25] is employed to identify various sources of errors, which are displayed in Figure 17. Using the improved algorithm, the noise and temperature characteristics of the accelerometer have been significantly improved, in which the acceleration random walk is reduced from 0.0047 g/√h to 0.0036 g/√h and the zero instability is reduced from 0.17 g/h to 0.13 g/h.

## 5. Conclusions

In this paper, a fusion algorithm for temperature compensation is studied to increase the accuracy of a High-G accelerometer. Firstly, the accelerometer signal is decomposed into five IMFs using VMD. Next, the IMFs are segmented by FE, a pure noise component is removed, the mixing component is denoised by wavelet thresholding, and a model of the drift component is established through PSO-BP. Signal reconstruction is finally put into practice and the temperature experiment is carried out. From the results, it can be seen that the temperature coefficient changes from 2.4 × 10^−4^ g/°C to 1.8 × 10^−5^ g/°C, and Allan variance is employed to compare the outcomes. The results indicate that the acceleration random walk and zero-deviation stability change from 0.0047 g/√h and 0.17 g/h to 0.0036 g/√h and 0.13 g/h, respectively. The fusion algorithm is shown to be the best, which implies that the proposed algorithm can suppress the noise effectively and have a high-precision compensation simultaneously. 

## Figures and Tables

**Table 1:** Optimized structural parameters of the accelerometer.

Parameter	Size	Parameter	Size
Device thickness (μm)	60	Number of movable comb teeth (single side)	26
Mass block mass (μg)	560	Horizontal axis return beam size (μm)	800 × 15
Comb gap (μm)	3	Blocking block clearance (μm)	2
Comb size (μm)	300 × 8		

**Table 2:** The first six-order modal frequencies of the accelerometer.

Modal Order	1	2	3	4	5	6
Frequency (Hz)	8263.3	11,027	12,752	20,034	22,640	53,017

## Data Availability

The data presented in this study are available on request from the corresponding author.
